# Nurse‐Led Patient‐Reported Outcome Assessment of Symptom Burden and Quality of Life in Myeloproliferative Neoplasms: An Observational Study

**DOI:** 10.1002/nop2.70784

**Published:** 2026-08-30

**Authors:** Daniela Berardinelli, Carmen Fava, Valentina Bonuomo, Selene Grano, Federica Sinisi, Inga Ventimiglia, Graziella Costamagna, Paola Di Giulio, Sara Campagna, Andrea Ricotti, Valerio Dimonte

**Affiliations:** ^1^ Department of Public Health and Pediatrics University of Torino Torino Italy; ^2^ Department of Clinical and Biological Sciences University of Torino Torino Italy; ^3^ AO Ordine Mauriziano Hospital Torino Italy

**Keywords:** myeloproliferative neoplasms, nurse‐led care, patient‐centred care, patient‐reported outcome measures, symptom burden

## Abstract

**Aim:**

To investigate the symptom burden associated with MPNs and its impact on patients' psychophysical well‐being and quality of life through a structured PROM‐based nurse‐led visit.

**Design:**

Observational study.

**Methods:**

We included MPN patients followed at the haematology outpatient clinic of a large university‐affiliated hospital in Italy. Data were collected from September 2024 to March 2025 on 102 patients who underwent a structured PROM‐based nurse‐led visit supported by a dedicated symptom diary.

**Results:**

Symptom burden was substantial (85% were symptomatic) across all disease subtypes, with fatigue emerging as the most prominent symptom, affecting 72% of patients and accompanied by marked psychological distress (42% and 44% with anxious and depressive symptoms, respectively). Overall health‐related quality of life was predominantly moderate (mean score: 70.7 ± 21.3). Nearly all moderate‐to‐severe symptoms, including fatigue, inactivity, pain, itching, and abdominal discomfort, were strongly associated with impaired global health. 43% of patients need support in becoming more proactive in managing their disease, and satisfaction with the visit was very high (mean score 9.3 ± 1.3/10).

**Conclusion:**

The impact of MPNs was significant and multifaceted, affecting patients' physical, emotional, and social well‐being. Structured PROM‐based nurse‐led visits play a key role in detecting patients' needs and supporting symptom management across all domains of well‐being, fostering motivated engagement.

**Implications for the Profession and Patient Care:**

Integrating structured PROM‐based nurse‐led visits into routine haematology care may improve symptom monitoring, support patient engagement, and guide timely and tailored supportive interventions.

**Impact:**

- Patients with MPNs experience a substantial physical and psychosocial symptom burden.
- Symptom clusters are associated with anxiety, depression, and reduced quality of life.
- Structured PROM‐based nurse‐led visits may facilitate the early identification of unmet supportive care needs.

**Reporting Method:**

Guidelines for reporting observational studies (STROBE):

**Patient Contribution:**

Patients actively participated during the nurse‐led visit and in data collection.

## 1 Introduction

Myelofibrosis (MF), polycythaemia vera (PV), and essential thrombocythaemia (ET) are Philadelphia chromosome–negative Myeloproliferative Neoplasms (MPNs) commonly associated with *JAK2*, *CALR*, or *MPL* driver mutations (Thiele et al. 2023). These are rare disorders characterised by an increased risk of thrombosis and cardiovascular events (Thiele et al. 2023). Major causes of morbidity and mortality include thrombohaemorrhagic complications, disease transformation into secondary MF, or progression to acute myeloid leukaemia (Thiele et al. 2023).

Symptom profiles differ by subtype but frequently include fatigue and a marked reduction in quality of life (QOL) (Caocci et al. 2025; Eppingbroek et al. 2024). Other common symptoms include pruritus, night sweats, insomnia, headaches, cognitive difficulties, and manifestations related to splenomegaly, such as early satiety, abdominal pain, or discomfort (Poullet et al. 2025). Targeted therapies have improved symptom burden (Mesa et al. 2023); however, assessing QOL and selecting appropriate symptom‐directed interventions remains challenging, as routine clinical evaluation alone may not fully capture the multidimensional impact of MPNs on patients' daily lives. This highlights the need for complementary patient‐reported assessments.

## 2 Background

Systematic integration of patient‐reported outcome measures (PROMs) into clinical practice can improve outcomes, particularly screening, monitoring, and the tailoring of supportive interventions in cancer‐related conditions (Bonsel et al. 2024). PROMs also enhance patient–clinician communication, engagement, and activation in disease management (Watson et al. 2021). They are particularly valuable in rare diseases, capturing patients' lived experiences and aspects of living with the disease that are most meaningful to them (Aiyegbusi et al. 2024). Yet, their routine use remains limited (Huberts et al. 2024).

A person‐centred care (PCC) approach is essential in chronic haematological malignancies to address disease burden and psychosocial challenges.

The systematic use of PROMs supports the therapeutic relationship and integration of patient preferences into care planning (Watson et al. 2021). However, haematology practice still tends to focus predominantly on biomedical outcomes. Although not routinely embedded in daily haematology practice, PROMs are recognised as useful communication tools for identifying areas requiring targeted interventions (Lai et al. 2021). Symptom diaries represent a valuable method for capturing PROMs data, allowing the monitoring of variations in symptom intensity and perception (IJzerman‐Korevaar et al. 2021); patients perceive them as helpful for reducing recall bias, monitoring changes over time, and facilitating communication with their clinician (Campbell et al. 2022). Data on PROMs addressing psychological health and well‐being in MPNs remain scarce (Bao et al. 2023).

In this context, nurses play an important role in comprehensive symptom assessment, patient education, and supportive care. Their close and continuous interaction with patients makes nurse‐led visits particularly suitable for integrating PROMs into routine outpatient practice and facilitating communication within the multidisciplinary team (Watson et al. 2021). Nurse‐led clinics, in which nurses with advanced competencies provide autonomous, evidence‐based, and multidisciplinary care, are increasingly recognised as an important model for the management of patients with chronic conditions. Their activities typically include a nurse‐led visit based on a comprehensive symptom assessment, patient education, counselling, care coordination, and support for self‐management (Terry et al. 2024). By fostering continuous patient monitoring and communication, nurse‐led visits provide an appropriate setting for the routine integration of PROMs into clinical practice and for the identification of patients requiring additional supportive interventions. In patients with chronic haematological malignancies, promoting self‐management and active patient engagement is particularly relevant, given the long‐term nature of the disease and the increasing use of oral targeted therapies.

Limited evidence is available regarding the implementation of structured PROM‐based nurse‐led visits during outpatient consultations. Furthermore, little is known about how multidimensional PROM‐based assessment may support the identification of patients with greater psychosocial needs in routine clinical practice. Given the substantial symptom burden and psychosocial challenges experienced by patients with MPNs, and the limited routine integration of PROMs into haematology care, this study aimed to evaluate symptom burden and its impact on psychosocial well‐being, QOL, and patient engagement through a structured PROM‐based nurse‐led visit.

## 3 Methods

### 3.1 Study Design

This observational study, incorporating a structured PROM‐based nurse‐led visit was conducted from September 2024 to February 2025 at a university‐affiliated haematology outpatient clinic in Italy. The clinic provides diagnosis, treatment, and follow‐up care for MPN patients.

### 3.2 Setting and Participants

Eligible participants were adults (≥ 18 years) diagnosed with ET, PV, or MF according to the 5th edition of the World Health Organisation (WHO) Classification of Haematolymphoid Tumours. Only patients receiving pharmacological treatment for MPNs were included as this group is considered at higher risk of disease‐related complications and requires closer clinical and laboratory monitoring. Additional inclusion criteria were the ability to communicate in Italian; absence of significant cognitive impairment; and willingness to complete PROMs with minimal assistance. Eligible patients were identified by physicians during outpatient visits and informed by a nurse who explained the study purpose and procedures.

A consecutive sampling strategy was adopted, and all eligible patients attending the outpatient clinic during the study period were invited to participate. No eligible patients declined participation or were excluded after screening.

### 3.3 Procedure and Measures

#### 3.3.1 General and Disease‐Related Characteristics

Information on age, sex, BMI, blood pressure, concomitant medications, clinical history, MPN‐related data (subtype, disease duration, prognostic risk, current therapies, and caregiver presence) was obtained during the nurse‐led visits and from electronic medical records. Prognostic risk was classified according to the NCCN Guidelines Version 1 2026 for MPNs, using DIPSS (Dynamic International Prognostic Scoring System) and MYSEC‐PM (MF Secondary to PV and ET‐Prognostic Model) for MF, the Conventional Risk Model for PV, and the IPSET (International Prognostic Score of Thrombosis in WHO–ET) score for ET (NCCN Guidelines Version 1 2026 on Myeloproliferative Neoplasms, 2026).

### 3.4 Data Collection

#### 3.4.1 Nurse‐Led Visit and Patient‐Reported Outcomes (PROs) Measures

The outpatient clinic follows approximately 200 patients with MPNs (around 20 new diagnoses per year), of whom 102 met the eligibility criteria and were enrolled.

Data collection occurred during a structured PROM‐based nurse‐led visit specifically implemented for this study. During the study, the visit took place immediately before the physician's consultation and lasted up to 1 h. During the visit, the nurse introduced a dedicated symptom diary, explained its contents, and supported patients in completing PROMs according to their own perceptions and experiences. The diary consisted of a compilation of validated PROM instruments assessing physical symptoms, fatigue, nutritional risk, medication adherence, anxiety, depression, QOL, work productivity, patient activation and satisfaction with care (Box 1). The symptom diary, printed in A4 format, included a section with self‐care recommendations. The nurse reviewed patient responses, facilitated discussion on reported concerns and provided individualised education and practical suggestions to support symptom control, self‐care, and self‐management. Rather than representing a simple questionnaire administration, the nurse‐led visit involved active listening, review of patients' responses, and discussion of reported concerns, occupying most of the visit. Relevant information from the diary was shared with the treating physician at the end of the nurse‐led visit.

BOX 1 **Tools description for capturing PROMs during the nurse‐led visit.**

**Table:** 

Tools	Description
Myeloproliferative Neoplasm Symptom Assessment Form Total Symptom Score (**MPN‐SAF TSS**)^a^	The MPN‐SAF TSS is a self‐report instrument, consisting of 10 items assessing the presence and severity of symptoms such as fatigue, early satiety, concentration problems, abdominal discomfort and bone pain, the week preceding the assessment, on a scale ranging from 0 (absent) to 10 (worst imaginable). The mean score of all 10 items is then calculated. MPN symptom burden is defined as ‘mild’ if score ≤ 3, moderate if ≥ 4 and ≤ 6 and severe if ≥ 7.
Functional Assessment of Chronic Illness Therapy‐Fatigue Scale **(FACIT‐Fatigue‐10)** ^b^	The FACIT‐Fatigue‐10 is a 10‐item self‐report questionnaire assessing tiredness, weakness, and difficulty in performing usual activities due to fatigue in the last 7 days. All items are rated on a 5‐point Likert scale, ranging from 1 (not at all) to 5 (very much); the overall score ranges from 10 to 50, with higher scores denoting higher levels of fatigue.
Medication Adherence Report Scale (**MARS‐5i**)^c^	The MARS‐5i Italian version is a 5‐item self‐report questionnaire that investigates the causes and frequency of non‐adherence to drug therapy. The score is calculated by summing the scores (range 1–5) of each question and ranges from 5 to 25. A higher score indicates better medication adherence.
Malnutrition Screening Tool (**MST**)^d^	The MST is a two‐question malnutrition screening tool that identifies adults malnourished or at risk of malnutrition, and could benefit from nutritional support. A score ≥ 2 indicates a risk of malnutrition.
Generalised Anxiety Disorder– 7 (**GAD‐7**)^e^	GAD‐7 is a screening instrument for generalised anxiety disorder with clinical diagnostic value. The scale consists of seven questions assessed on a 4‐point Likert response scale, from 0 = Never to 3 = Almost every day, resulting in a score between 0 and 21. A value of 10 was identified as the cut‐off for a clinical diagnosis with good sensitivity and specificity (over 80%).
Patient Health Questionnaire—9 (**PHQ‐9**)^f^	PHQ‐9 is an instrument that enables screening, diagnosis, monitoring and measuring of the severity of depression. It consists of 9 items that correspond to the symptoms of major depression according to the DSM‐IV. The score ranges between 0 and 27. Scores between 0 and 9 indicate subthreshold depression. A score of 10 is indicated as the point at which the sensitivity and specificity of the instrument are recognised as optimal for detecting clinically relevant depression (5–9 = Minimal depressive symptoms / subthreshold depression, 10–14 = Minor depression / Mild major depression, 15–19 = Moderate major depression, ≥ 20 = Severe major depression).
QUALITY OF LIFE (EORTC‐ QLQ‐F17) ^g^	The EORTC QLQ‐F17 is a multidimensional measure of QoL. It consists of 17 items and includes five functional scales (physical, role, emotional, social, and cognitive) and one on health status/global QoL. The questions refer to the last 7 days. The raw scores are transformed into a linear scale ranging from 0 to 100, with higher scores indicating a higher level of functioning and health status/QoL.
Work and Activity Productivity Impairment Questionnaire: General Health, v2.3 **(WPAI:GH**)^h^	The WPAI:GH is a self‐report questionnaire of 6 questions assessing the effects of health problems on the ability to work and carry out normal daily activities. Health problems are defined as any physical or psychological disorder or issue occurred in the last 7 days. Questions include employment status, the number of working hours lost due to health problems or other reasons, the actual hours worked, the impact of the illness on the ability to work, and on daily activities. It produces four sub‐scores: work time lost due to health issues (absenteeism), impairment at work (reduced on‐the‐job effectiveness), overall work impairment (work productivity loss), and activity impairment (impairment of daily activities). The sub‐scores are transformed into impairment percentages (range from 0 to 100) with higher numbers indicating greater impairment and less productivity.
Patient Activation Measure (**PAM13‐I**)i^i^	The PAM13‐I is a self‐report questionnaire, validated in Italian, with 13 items on a Likert scale with scores from 1 to 5: (1) ‘Strongly disagree’, (2) ‘Disagree’, (3) ‘Agree’, (4) ‘Strongly agree’ and (5) ‘Not applicable’. The PAM13‐I reflects the four stages of activation of the individual in the management of his or her disease, with a progressive difficulty of the items: level 1 (not believing activation important), level 2 (a lack of knowledge and confidence to take action), level 3 (beginning to take action) and level 4 (taking action). A higher PAM13‐I score indicates a better level of patient activation.
Satisfaction	Patients completed a questionnaire to assess their satisfaction with the nurse‐led visit at its conclusion. Satisfaction was measured using a 0–10 visual analogue scale (VAS), with higher scores indicating greater satisfaction. Patients' caregiver involvement during the nurse‐led visit was guaranteed based on patients will.

^a^ Scherber, R., Dueck, A. C., Johansson, P., Barbui, T., Barosi, G., Vannucchi, A. M., Passamonti, F., Andreasson, B., Ferarri, M. L., Rambaldi, A., Samuelsson, J., Birgegard, G., Tefferi, A., Harrison, C. N., Radia, D., & Mesa, R. A. (2011). The Myeloproliferative Neoplasm Symptom Assessment Form (MPN‐SAF): International prospective validation and reliability trial in 402 patients. Blood, 118(2), 401–408. https://doi.org/10.1182/blood‐2011‐01‐328,955.

^b^ PROMIS SF v1.0 Fatigue 10a. (n.d.). FACIT Group. Retrieved April 28, 2025, from https://www.facit.org/measures/promis‐sf‐v1.0‐fatigue‐10a.

^c^ SOLE Study Group of(2019). Translation and initial validation of the Medication Adherence Report Scale (MARS) in Italian patients with Crohn's Disease. Digestive and Liver Disease: Official Journal of the Italian Society of Gastroenterology and the Italian Association for the Study of the Liver, 51(5), 640–647. https://doi.org/10.1016/j.dld.2018.09.026.

^d^ Ferguson, M., Capra, S., Bauer, J., & Banks, M. (1999). Development of a valid and reliable malnutrition screening tool for adult acute hospital patients. Nutrition (Burbank, Los Angeles County, Calif.), 15(6), 458–464. https://doi.org/10.1016/s0899‐9007(99)00084‐2.

^e^ Spitzer, R. L., Kroenke, K., Williams, J. B. W., & Löwe, B. (2006). A brief measure for assessing generalised anxiety disorder: The GAD‐7. Archives of Internal Medicine, 166(10), 1092–1097. https://doi.org/10.1001/archinte.166.10.1092.

^f^ Kroenke, K., Spitzer, R. L., & Williams, J. B. (2001). The PHQ‐9: Validity of a brief depression severity measure. Journal of General Internal Medicine, 16(9), 606–613. https://doi.org/10.1046/j.1525‐1497.2001.016009606.x.

^g^ Quality of Life Core Function Questionnaire. (2023, August 17). EORTC—Quality of Life. https://qol.eortc.org/questionnaire/qlq‐fa12‐2/.

^h^ Reilly, M. C., Zbrozek, A. S., & Dukes, E. M. (1993). The validity and reproducibility of a work productivity and activity impairment instrument. PharmacoEconomics, 4(5), 353–365. https://doi.org/10.2165/00019053‐199304050‐00006.

^i^ Graffigna, G., Barello, S., Bonanomi, A., Lozza, E., & Hibbard, J. (2015). Measuring patient activation in Italy: Translation, adaptation and validation of the Italian version of the patient activation measure 13 (PAM13‐I). BMC Medical Informatics and Decision Making, 15, 109. https://doi.org/10.1186/s12911‐015‐0232‐9.

All participants completed the symptom diary during the nurse‐led visit, and no missing data were recorded. Patients were then asked to bring the symptom diary to their next follow‐up visit.

The nurse‐led visits were conducted by a nurse with advanced academic training (master's degree and doctoral training) and specific expertise in haematology, including training in the administration and interpretation of PROMs.

### 3.5 Ethical Considerations

The study complied with the Declaration of Helsinki and the International Conference on Harmonisation guidelines for Good Clinical Practice. Approval was obtained by the ethics committee of the participating site (‘Comitato Etico Territoriale Interaziendale A.O.U. Città della Salute e della Scienza di Torino’, approval no 307/2024) and written informed consent was obtained from all participants.

### 3.6 Statistical Analysis

Data were collected and managed using REDCap electronic data capture tools hosted at Hospital A.O Ordine Mauriziano. All statistical analyses were conducted using R version 4.3.3. Continuous variables were described as mean and standard deviation (SD) if normally distributed, or as median and interquartile range (IQR) if not normally distributed. Categorical variables were summarised as absolute frequencies and percentages. Differences between groups were estimated using the standardised mean difference (SMD) for normally distributed continuous and categorical variables, and Cliff's delta for non‐normally distributed continuous variables. SMD values of 0.2, 0.5, and 0.8 were considered to represent small, medium, and large differences, respectively. For Cliff's delta (*δ*), the magnitude was assessed according to these thresholds: |*δ*| < 0.147 indicating a negligible effect, 0.147 ≤ |*δ*| < 0.33 a small effect, 0.33 ≤ |*δ*| < 0.474 a medium effect, and |*δ*| ≥ 0.474 a large effect. Univariate logistic regression analyses were performed to assess the association between individual predictors and the outcome. A *p* < 0.05 was considered statistically significant. Due to the limited sample size, multivariable models were not considered appropriate, as the available sample did not allow reliable adjustment for multiple potential confounding variables.

## 4 Results

Of the 200 patients followed, 102 were receiving active pharmacological treatment and were therefore eligible and enrolled between September 2024 and March 2025. The mean age of the participants was 69 (± 12.4) years, with the majority being women (56%). ET was the most prevalent (60%), followed by MF (20%) and PV (20%). Median time since diagnosis was 3 years (IQR, 1–8 years), with a range of less than 1 year to 31 years. All patients were treated with pharmacological therapies, the most common was hydroxyurea; 26 patients were full or part‐time employed (Table 1).

**TABLE 1:** Sociodemographic and disease‐related characteristics of MPN patients.

	ALL (*n* = 102)	ET (*n* = 62, 60%)	MF (*n* = 20, 20%)	PV (*n* = 20, 20%)		
	*n* (%)	*n* (%)	*n* (%)	*n* (%)	Δ	Difference
Female	57 (55.9)	35 (56.4)	12 (60.0)	10 (50.0)	0.13	No difference
Age (Median, IQR)*	70 (61–79)	68 (60–79)	78 (67–80)	73 (64–79)	0.12	No difference
Employment status						
Employed	26 (25.4)	21 (33.9)	1 (5.0)	4 (20.0)	**0.52**	**Medium**
Time from diagnosis, years (Median, IQR)*	3 (1–8)	2 (0.9–8)	4.6 (2.7–4)	2.1 (1–6.25)	0.28	Small
Comorbidities						
Cardiovascular	59 (57.8)	35 (56.4)	11 (55.0)	13 (65.0)	0.14	No difference
Endocrine	27 (26.5)	18 (29.0)	4 (20.0)	5 (25.0)	0.14	No difference
Gastrointestinal	17 (16.7)	11 (17.4)	3 (15.0)	3 (15.0)	0.05	No difference
Oncological	15 (14.7)	8 (12.9)	4 (20.0)	3 (15.0)	0.13	No difference
Pulmonary	9 (8.8)	5 (8.0)	1 (5.0)	3 (15.0)	0.23	Small
Gynaecological and urological	8 (7.8)	7 (11.3)	0	1 (5.0)	0.35	Small
Neurological	8 (7.8)	4 (6.4)	2 (10.0)	2 (10.0)	0.09	No difference
Rheumatological‐autoimmune	7 (6.8)	6 (9.6)	1 (5.0)	0	0.32	Small
Psychiatric	8 (7.8)	7 (11.3)	0	1 (5.0)	0.22	Small
Others	15 (14.7)	8 (12.9)	1 (5.0)	5 (25.0)	0.39	Small
Prognostic risk						
Low	35 (34.3)	25 (40.3)	10 (50)	0		
High	67 (65.7)	37 (59.7)	10 (50)	20 (100)	**0.92**	**Large**
Current therapy						
Hydroxyurea	61 (59.8)	43 (69.3)	6 (30.0)	12 (60.0)	**0.56**	**Medium**
Jak Inhibitor	19 (18.6)	1 (1.6)	13 (65.0)	5 (25.0)	**1.14**	**Large**
Interferon	14 (13.7)	13 (20.9)	0	1 (5.0)	**0.51**	**Medium**
Anagrelide	4 (3.9)	4 (6.45)	0	0	0.25	Small
Other medications	7 (6.9)	1 (1.6)	4 (20.0)	2 (10.0)	0.42	Small
Concomitant medications (Median, IQR)*	5 (3–7)	5 (2–7)	5 (3–7)	5 (3–6)	0.15	Small

*Note:* Δ: SMD (standardised mean difference, used to estimate effect size for normal continues and ordinal data). Bold values indicate medium or large effect sizes.

* Cliff's delta (used to estimate effect size non‐normal continuous data).

The large majority (85%) of patients were symptomatic, presenting a median MPN‐SAF TSS score of 14.5 (IQR, 6–27.2) (Table 2); 15 (14.7%) patients showed no symptoms, whereas 13 (12.7%) had exclusively mild symptoms (rated as < 4), with a range of 1–6 symptoms for each patient. The median TSS was comparable across MPN subtypes (Δ 0.17, small difference).

**TABLE 2:** Assessment of psycho‐physical symptom burden, malnutrition risk, activation and medication adherence by disease type.

	ALL (*n* = 102)	ALL (*n* = 102)	ET (*n* = 62)	MF (*n* = 20)	PV (*n* = 20)		
	*n* (%)	*n* (%)	*n* (%)	*n* (%)	*n* (%)	Δ	Difference
Symptom Burden (number of symptoms)							
0	15 (14.7)		10 (16.1)	3 (15.0)	2 (10.0)	0.37	Small
1	13 (12.7)		9 (14.5)	3 (15.0)	1 (5.0)		
2–3	28 (27.4)		17 (27.4)	6 (30.0)	5 (5.0)		
4–6	37 (36.2)		20 (32.3)	7 (35.0)	10 (50.0)		
> 6	9 (8.8)		6 (9.7)	1 (5.0)	2 (10.0)		
MPN‐SAF symptoms		**Severity** > **3**					
Fatigue	74 (72.5)	57 (55.9)	43 (69.3)	16 (80.0)	15 (75.0)	0.16	No difference
Inactivity	52 (50.9)	37 (36.3)	29 (46.8)	13 (65.0)	10 (50.0)	0.14	No difference
Abdominal discomfort	41 (40.2)	30 (29.4)	22 (35.5)	9 (45.0)	10 (50.0)	0.10	No difference
Concentration problems	38 (37.2)	25 (24.5)	24 (38.7)	6 (30.0)	8 (40.0)	0.13	No difference
Early satiety	36 (35.3)	23 (22.5)	21 (33.9)	5 (25.0)	10 (50.0)	0.26	Small
Bone pain	36 (35.3)	28 (27.4)	19 (30.6)	8 (40.0)	9 (45.0)	0.19	No difference
Itching	26 (25.5)	20 (19.6)	16 (25.8)	2 (10.0)	8 (40.0)	0.38	Small
Night sweats	17 (16.7)	14 (13.7)	10 (16.1)	3 (15.0)	4 (20.0)	0.10	No difference
Weight loss	17 (16.7)	8 (7.8)	8 (12.9)	2 (10.0)	7 (35.0)	0.37	Small
Fever	2 (1.9)	2 (1.9)	2 (3.2)	0	0	0.17	No difference
MPN‐SAF TSS (Median, IQR)*	14.5 (6–27.2)		12 (3–24.7)	14.5 (5.7–24.2)	21 (9–29.5)	0.17	Small
≥ 2 Moderate‐to‐severe symptoms (Score > 3)	57 (55.8)		31 (50)	12 (60)	14 (70)	0.28	Small
Facit‐fatigue 10 (Mean, SD)	47.9 (7.4)		47.5 (2.4)	48.9 (2.3)	48.2 (2.2)	0.14	No difference
Malnutrition risk	12 (11.8)		7 (11.3)	1 (5)	4 (20)	0.31	Small
Anxiety							
None (Score 0–4)	59 (57.8)		35 (56.4)	13 (65)	11 (55)	0.26	Small
Mild (Score 5–9)	23 (22.5)		14 (22.0)	3 (15)	6 (30)		
Clinical anxiety (≥ 10)	20 (19.6)		13 (21.0)	4 (20.0)	3 (15.0)		
Total (Median, IQR)*	3.5 (0.25–8)		3 (0.25–9)	3 (0–8)	4 (1.75–8)	0.10	No difference
Depression							
None (Score 0–4)	58 (56.8)		36 (58.1)	10 (50)	12 (60)	0.50	Medium
Mild (Score 5–9)	32 (31.4)		17 (27.4)	10 (50)	5 (25)		
Clinical depression (≥ 10)	12 (11.8)		9 (14.5)	0	3 (15)		
Total (Median, IQR)*	4 (2–7)		3 (1–6.75)	4.5 (2–7.25)	4 (2–7.25)	0.01	No difference
Patient engagement							
Inactive (Level 1–2)	44 (43.1)		23 (37.1)	11 (55)	10 (50)	0.24	Small
Active (Level 3–4)	58 (56.9)		39 (63.0)	9 (45)	10 (50)		
Medication adherence (Median, IQR)*	25 (24–25)		25 (25–25)	25 (24.8–25)	25 (24–25)	0.19	Small

*Note:* Δ indicates standardised mean difference (SMD) for normally distributed continuous and ordinal variables.

* Cliff's delta was used as the effect size measure for non‐normally distributed continuous variables.

Although most symptoms were physical, 38 individuals (37.2%) also reported cognitive complaints, such as problems with concentration. Fatigue was the most frequent symptom (72%). Overall, 57 patients rated their fatigue as moderate‐to‐severe (score > 3) (Table 2), including two‐thirds of those with ET (30/43), the majority of MF patients (12/16), and all PV patients who reported fatigue (15/15). Fatigue was classified as moderate in 28 patients and severe (> 6) in 29. Among the 57 patients who reported moderate‐to‐severe fatigue, 41 also reported inactivity, which was experienced by half of the study population (52/102). The other most prevalent physical symptoms were abdominal discomfort and early satiety (likely associated with splenomegaly), reported by 41 (40.2%) and 36 patients (35.3%), respectively, and predominantly among those with PV (10 patients, 50%). Some symptoms, though less frequent, were disturbing, such as bone pain, described as diffuse and unrelated to arthritis or joint disease, experienced by 36 individuals (35.3%). Most rated it as moderate‐to‐severe (28/36 patients). Although only 26 patients reported itching, it was rated as severe (score ≥ 7) by 20 of them (12 with ET, 2 with MF, and 6 with PV). Night sweats, weight loss, and fever were among the least frequently reported symptoms (Table 2).

**FIGURE 1:**
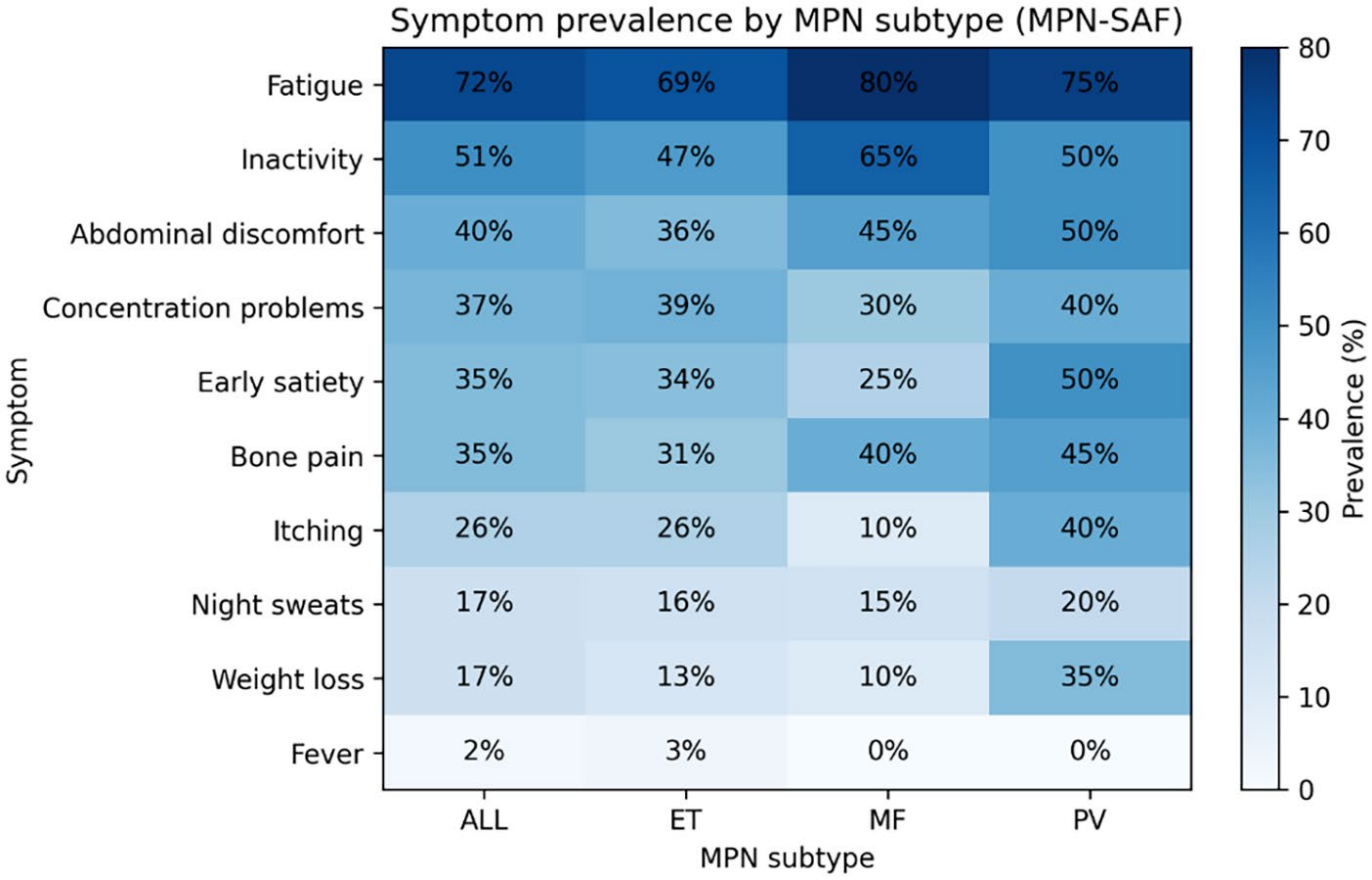
Prevalence of MPN‐related symptoms by disease subtype.

The heatmap (Figure 1) showed the prevalence (%) of MPN‐related symptoms across disease subtypes (ET, MF, PV).

Patients with PV reported the highest symptom scores, with a median TSS of 21 (IQR, 9–29.5), compared to 12 (IQR, 3–24.7) in patients with ET and 14.5 (IQR, 5.7–24.2) in those with MF (Table 2). Women also had a higher median TSS score of 19 (IQR, 8–31), compared with 10 (IQR, 3–22) in men (Δ 0.22, small difference).

Prognostic risk was not associated with symptom burden; patients classified as low risk reported a median of 4 symptoms (IQR 1–6), compared with 3 (IQR 2–5) among high‐risk patients (Δ 0.04, no difference).

According to the MST, the risk of malnutrition was generally low; 12 patients were identified as being at risk, of which 4 had PV.

Nearly all patients with moderate‐to‐severe clinical depression (11/12) reported at least two moderate‐to‐severe symptoms (> 3). Among patients reporting at least two moderate‐to‐severe symptoms, about half (29/57, 50.9%) had subthreshold depressive symptoms. All the patients without symptoms showed no depression.

Almost half of the patients (*n* = 45) had a low level of engagement and proactivity in maintaining their health and making decisions regarding their care (score 1 or 2 on the PAM‐13 questionnaire); however, adherence to haematological treatment was generally excellent. Only two patients reported lower medication adherence and both had a low engagement level.

Overall work impairment was considerable (WPAI median 18.4%, IQR 0%–69.6%; mean 34.5% ± 38.3%).

Satisfaction with the nurse‐led visit was high, with a mean score of 9.3 ± 1.3 on a 10‐point scale. Five patients rated the visit below 8, including one patient who gave a satisfaction score of 1. This patient had a high symptom burden (four symptoms > 4, including fatigue rated 7), moderate anxiety, severe depression, poor QOL (50/100), and the lowest PAM level.

The overall QOL was moderate‐to‐good, with a mean score of 70.7 ± 21.3 (Table 3). Patients with ET reported better QOL (mean score 74.2 ± 22.9) compared to those with MF (62.1 ± 19.4) or PV (68.3 ± 15.4). Notably, although PV patients reported a greater number and severity of symptoms, their mean QOL score was higher than that of MF patients.

**TABLE 3:** Health‐related quality of life scores of MPN patients by disease type, number of symptoms and moderate to severe fatigue.

Qlq‐F17 Scores (Mean, SD)	ALL (*N* = 102)	ET (*n* = 62)	MF (*n* = 20)	PV (*n* = 20)	Δ	Difference
Physical functioning	78.9 (19.6)	81.4 (19.7)	74.3 (22.6)	76.0 (15.3)	0.24	Small
Role functioning	80.9 (27.1)	83.4 (24.7)	80 (25.7)	74.2 (34.8)	0.21	Small
Emotional functioning	77.8 (24.2)	76.9 (25.7)	81.7 (19.9)	76.7 (23.8)	0.15	No difference
Social functioning	87.4 (21.9)	86.3 (24.1)	88.3 (18.8)	90 (18.2)	0.12	No difference
Cognitive functioning	85.6 (22.5)	83.9 (25.6)	88.3 (15.4)	88.3 (18.01)	0.14	No difference
Global health	70.7 (21.3)	74.2 (22.9)	62.1 (19.4)	68.3 (15.4)	0.41	Small
Global health with *n* symptoms						
0 (*N* = 15)	83.9 (17.1)	84.2 (± 20.9)	80.5 (± 4.8)	87.5 (± 5.9)	**0.58**	**Medium**
1 (*N* = 13)	76.9 (17.1)	81.48 (± 17.1)	61.1 (± 9.6)	83.3 (−)*	**1.47**	**Large**
2–3 (*N* = 28)	73.5 (19.4)	73.0 (± 22.9)	70.8 (± 16.5)	78.3 (± 7.4)	0.34	Small
4–6 (*N* = 37)	63.7 (20.2)	68.7 (± 23.4)	53.6 (15.9)	60.8 (± 13.1)	**0.56**	**Medium**
> 6 (*N* = 9)	59.3 (30.2)	68.1 (± 30.5)	16.7 (−)*	54.2 (± 17.7)	**0.56**	**Medium**
With fatigue severity > 3	63.4 (±21.8)	67.2 (± 25.3)	54.2 (± 18.3)	63.3 (± 14.4)	0.45	Small

*Note:* Δ indicates standardised mean difference (SMD) for normally distributed continuous and ordinal variables. Bold values indicate medium or large effect sizes.

* Values reported by one patient.

The relationship between the number of symptoms and global health QOL score was explored (Table 3): asymptomatic patients generally showed a better overall QOL score which decreased as the number of symptoms increased.

QOL was comparable between male (70.9 ± 19.4) and female patients (70.5 ± 22.9) (Δ 0.02, no difference) with no differences between low‐ and high‐risk clinical groups (68.6 ± 24.8 vs. 71.8 ± 19.5) (Δ 0.15, no difference).

The impact of fatigue on QOL was specifically assessed in patients reporting moderate‐to‐severe fatigue (> 3) compared with those with mild fatigue (Table 3). Fatigue negatively affected QOL across all patients, with the greatest impact in those with MF, who had a mean QOL score of 54 (Δ 0.45, small difference). Patients reporting low fatigue levels (< 4) had a mean global health score of 79.8 ± 17.0.

In general, emotional and physical functioning obtained the lowest scores across all MPN subtypes compared with the other functional subscales of the EORTC QLQ‐F17. Overall, there was considerable variability in QOL assessments, with patients within each disease subtype showing a wide range of judgements from better to worse.

In the univariate analysis (Table 4), symptoms of moderate‐to‐severe intensity were significantly associated with impaired global health (EORTC QLQ‐F17 < 75), particularly fatigue (OR 4.7, 95% CI 2.1–11.4, *p* < 0.001), bone pain (OR 4.2, 95% CI 1.7–11.5, *p* = 0.003), and itching (OR 4.4, 95% CI 1.6–14.8, *p* = 0.008).

**TABLE 4:** Association of symptoms and psychosocial factors with impaired general health (EORTC QLQ‐F17 < 75), moderate‐to‐severe anxiety (≥ 10), and clinical depression (≥ 10) in patients with MPN.

Variables	Global health < 75 OR (95% CI), *P*	Anxiety ≥ 10 OR (95% CI), *P*	Depression ≥ 10 OR (95% CI), *P*	
Symptoms with severity > 3				
Fatigue	**4.7 (2.1–11.4), < 0.001**	1.6 (0.6–4.6), 0.362	**10.5 (1.9–196.3), 0.027**	Associated with impaired QOL & clinical depression
Inactivity	**3.1 (1.4–7.5), 0.008**	**3.4 (1.3–9.7), 0.017**	**11.6 (2.8–79.3), 0.002**	Associated with impaired QOL, clinical anxiety & depression
Abdominal discomfort	**2.6 (1.1–6.3), 0.036**	2.4 (0.8–6.6), 0.093	**6.2 (1.7–25.1), 0.006**	Associated with impaired QOL & clinical depression
Bone pain	**4.2 (1.7–11.5), 0.003**	2.0 (0.7–5.6), 0.176	**4.5 (1.3–16.8), 0.018**	Associated with impaired QOL & clinical depression
Concentration problems	1.1 (0.4–2.6), 0.914	**5.9 (2.1–17.5), 0.001**	**5.6 (1.6–20.9), 0.007**	Associated with clinical anxiety & depression
Early satiety	1.6 (0.6–4.2), 0.304	**2.9 (1.0–8.6), 0.043**	**10.0 (2.8–41.6), 0.001**	Associated with clinical anxiety & depression
Itching	**4.4 (1.6–14.8), 0.008**	2.1 (0.6–6.2), 0.198	**5.4 (1.5–19.8), 0.009**	Associated with impaired QOL & clinical depression
Night sweats	1.6 (0.5–5.2), 0.418	1.8 (0.4–6.2), 0.368	1.3 (0.2–5.7), 0.753	No significant association
≥ 2 symptom with severity > 3	**3.9 (1.7–9.3), 0.001**	**4.0 (1.3–14.9), 0.021**	**10.5 (1.9–196.3), 0.027**	Associated with impaired QOL, clinical anxiety & depression
Psychosocial outcomes				
Anxiety (≥ 10)	1.9 (0.7–5.4), 0.200	—	**8.3 (2.3–32.0), 0.001**	Associated with clinical depression
Clinical depression (≥ 10)	2.5 (0.7–9.9), 0.157	**8.3 (2.3–32.0), 0.001**	—	Associated with clinical anxiety
Patient engagement (active)	0.5 (0.2–1.2), 0.128	1.2 (0.4–3.4), 0.679	1.1 (0.3–4.0), 0.856	No significant association

*Note:* Odds ratios (OR) and 95% confidence intervals (CI) were estimated using univariate logistic regression models. Reference category for symptom variables: symptom severity ≤ 3. Reference category for patient engagement: inactive (PAM Levels 1–2). Reference categories for anxiety and depression: score < 10. Bold values indicate statistically significant associations (*p* < 0.05).

Moderate‐to‐severe physical and psychological symptoms, such as inactivity (OR 3.4, 95% CI 1.3–9.7, *p* = 0.017) and concentration problems (OR 5.9, 95% CI 2.1–17.5, *p* = 0.001), were significantly associated with moderate‐to‐severe anxiety. Similarly, moderate‐to‐severe fatigue (OR 10.5, 95% CI 1.9–196.3, *p* = 0.027), inactivity (OR 11.6, 95% CI 2.8–79.3, *p* = 0.002), and early satiety (OR 10.0, 95% CI 2.8–41.6, *p* = 0.001) were significantly associated with clinical depression. Overall, reporting at least two symptoms rated > 3 in severity was also associated with a significantly higher risk of impaired psychological and QOL outcomes (OR 3.9, 95% CI 1.7–9.3, *p* = 0.001).

Finally, patient engagement was not significantly associated with better general health or a lower risk of anxiety and depression symptoms.

## 5 Discussion

This study provides a comprehensive description of symptom burden in MPN patients and its impact on psychosocial well‐being and QOL through a structured PROM‐based nurse‐led visit and clinical assessment. While symptom prevalence aligns with previous studies (Eppingbroek et al. 2024; Poullet et al. 2025), our findings reinforce the substantial multidimensional burden experienced by patients in routine clinical practice and highlight the added value of integrating PROMs into nurse‐led care.

Consistent with other studies, fatigue was the most prevalent and burdensome symptom across all MPN subtypes, followed by inactivity and abdominal discomfort (Caocci et al. 2025; Khan et al. 2025; Manz et al. 2025). Symptom burden showed limited association with prognostic risk, confirming that biomedical risk scores, designed to estimate disease progression and survival, do not fully capture patients' lived experience (Poullet et al. 2025; Ritchie et al. 2022). Furthermore, because only patients receiving active treatment were included, symptom control achieved through therapy may have attenuated differences in symptom burden across risk groups. These findings further support the complementary role of PROMs in identifying unmet needs that may not be captured by conventional clinical assessments. Unlike most prior MPN studies, which typically focus on one or two domains, our integrated approach reflects the complexity of patients' needs in real‐world clinical settings. Moderate‐to‐severe symptoms rarely occur in isolation and were frequently accompanied by psychological vulnerability and functional impairment, underscoring the need for multidimensional assessment strategies.

Symptom patterns varied across MPN subtypes, as in previous studies (Caocci et al. 2025; Eppingbroek et al. 2024), with ET patients reporting fewer symptoms than those with PV and MF (Cherdchoo et al. 2023). Although MF is often associated with the greatest symptom burden, the higher overall TSS scores in PV patients observed in our cohort may reflect the limited sample size and the inherent heterogeneity of MPN populations (Caocci et al. 2025; Khan et al. 2025; Manz et al. 2025).

Women reported a higher symptom burden than men, with a median TSS score of 19 (IQR, 8–31), compared to 10 (IQR, 3–22), although QOL scores were comparable (Eppingbroek et al. 2024; Poullet et al. 2025). While a recent study by Eppingbroek et al. (2024) found no sex differences in QOL, an early study reported a lower QOL in women compared to men (Ritchie et al. 2022). This suggests that symptom perception and reporting may differ by gender, while adaptive mechanisms to chronic illness might moderate QOL differences (Ritchie et al. 2022). Recent evidence highlights the pivotal role of psychosocial adjustment and its influence on QOL in patients with MPN (Eppingbroek et al. 2025).

Overall, QOL was generally moderate to good, consistent with a comparable population reporting a slightly lower QOL (mean score 64) (Eppingbroek et al. 2024), but varied between each disease subtype with ET patients reporting better QOL compared to those with MF and PV (Ritchie et al. 2022).

Although increasing symptom burden was associated with reduced QOL, PV patients reported better QOL than MF patients despite higher symptom scores (Caocci et al. 2025). This finding suggests that patients' overall perception of QOL is influenced by multiple psychological, social, functional, and individual factors beyond symptom burden alone. In this context, PROMs provide a more comprehensive understanding of patients' experiences than symptom counts alone.

Psychological distress was common: approximately 1 out of 5 patients met criteria for clinical anxiety and 1 out of 8 for clinical depression, consistent with a previous study (Khan et al. 2025).

Nearly all moderate‐to‐severe symptoms were significantly associated with impaired global health and an increased risk of anxiety and depression. Fatigue, inactivity, early satiety, and concentration problems showed particularly strong associations with psychological distress. These findings extend previous work by identifying specific symptom clusters that may serve as early indicators of psychosocial risk. Mild anxiety (nearly a quarter) and depression (one third) were also frequent in oncology patient populations (Zeilinger et al. 2022). Although these individuals may not meet diagnostic criteria for clinical depression and anxiety, they represent a vulnerable group who may benefit from closer monitoring and supportive communication and timely referral when psychological distress persists or worsens to prevent progression to more severe conditions, including higher mortality (Huang et al. 2025). Routine PROM‐based assessment may help nurses identify these patients early and incorporate psychosocial needs into individualised care planning.

The symptom diary used in our clinic provided a structured approach to monitoring patients' health status. The benefits of symptom diaries measuring PROs are well documented (Campbell et al. 2022).

Another novel aspect of this study was the inclusion of patient activation as a component of a routine structured PROM‐based nurse‐led visit. Previous research using the PAM questionnaire reported slightly lower levels of activation compared to our cohort, further highlighting the need for structured, tailored support for this population (Eppingbroek et al. 2025). Nevertheless, the lack of a significant association between patient engagement and better general health or a lower risk of anxiety and depression symptoms may reflect the fact that patient engagement represents a distinct construct from psychological and physical well‐being. Patients may actively participate in the management of their disease while still experiencing emotional distress or perceiving poor health. Moreover, because patient engagement and psychological outcomes were assessed at a single time point, the study may not have fully captured their potentially dynamic relationship over time.

Our cohort demonstrated high adherence to haematological treatment; however, this finding suggests that medication adherence alone does not necessarily reflect active self‐management or psychological readiness to cope with a chronic malignancy. Targeted educational and motivational strategies therefore remain essential to enhance patients' knowledge, confidence, and self‐management skills.

### 5.1 Limits

Although no formal sample size calculation was performed, all consecutive eligible patients attending the outpatient clinic during the study period were included in the study. Nevertheless, the relatively limited sample size, particularly within some disease subgroups, reduced the possibility of performing multivariable analyses and may have limited the statistical power to detect subgroup differences.

Future studies including a larger and more diverse patient population are warranted. Nonetheless, one strength of this study is the systematic follow‐up of all the patients and the implementation of a multidisciplinary, patient‐centred model, enabling detailed analysis of symptom severity and its impact on QOL, as well as on anxiety and depression.

### 5.2 Implications for Practice

Non‐pharmacological approaches, including structured physical activity, have been explored for their potential to reduce cancer‐related fatigue and improve QOL, although evidence in the haematological population remains limited and sometimes inconsistent (Accogli et al. 2023). Nurses play a key motivational role in sustaining such interventions, for example, by encouraging patients to maintain and increase activity levels (Gyldenvang et al. 2025). Physical activity programmes delivered by multidisciplinary approaches or under nurse guidance have shown benefits in reducing fatigue and improving QOL (Mendizabal‐Gallastegui et al. 2023).

Nurses are ideally positioned to assess physical and psychological symptom burden, provide tailored clinical support, and support self‐care education. Comprehensive nurse‐led assessment may facilitate the early identification of patients experiencing increasing psycho‐physical burden, creating opportunities for timely supportive interventions and helping to address the sense of overwhelming often associated with chronic illness (Eppingbroek et al. 2025). Finally, oncology nurses are well positioned to support patients experiencing anxiety and depressive symptoms through active listening, empathetic communication, and timely referral when appropriate, while promoting patient engagement and adherence (Almutairi et al. 2025).

## 6 Conclusions

Overall, our findings suggest that the value of PROMs in MPN care extends beyond symptom measurement. Within a structured nurse‐led visit, PROMs may facilitate the identification of psychosocial distress, low patient activation, and complex symptom patterns that might otherwise remain unrecognised. This approach is particularly relevant for chronic haematological malignancies, where long‐term symptom management, psychological adjustment, and self‐care are central components of care. The integration of validated PROMs into routine outpatient care provides nurses with a structured approach to identifying unmet needs, facilitating communication, and supporting individualised symptom management. By fostering patient engagement and supporting self‐management, structured PROM‐based nurse‐led visits may contribute to more individualised and patient‐centred care. The high completion rate and absence of missing data underscore the acceptability of this approach for patients.

From an organisational perspective, these results suggest that a structured PROM‐based nurse‐led visit can be effectively incorporated into routine haematology care. To support broader implementation, investment in advanced training for haematology nurses should be provided, together with standardised PROM‐based assessment protocols and adequate staffing and protected time for structured nurse‐led consultations. Integrating PROMs into electronic health records and multidisciplinary workflows may further enhance continuity of care and facilitate timely intervention.

## Author Contributions

**Valentina Bonuomo:** formal analysis, investigation. **Inga Ventimiglia:** investigation, formal analysis. **Graziella Costamagna:** conceptualization. **Andrea Ricotti:** investigation, formal analysis, writing – original draft. **Carmen Fava:** conceptualization, methodology, writing – review and editing, supervision. **Federica Sinisi:** investigation, formal analysis. **Paola Di Giulio:** conceptualization, methodology, writing – review and editing, supervision. **Sara Campagna:** conceptualization. **Selene Grano:** investigation, formal analysis. **Valerio Dimonte:** supervision. **Daniela Berardinelli:** formal analysis, investigation, writing – original draft.

## Funding

The authors have nothing to report.

## Ethics Statement

Approval was obtained by the ethics committee of ‘A.O.U. Città della Salute e della Scienza di Torino’ (approval no 307/2022).There is a statistician on the author team (Andrea RICOTTI).

## Conflicts of Interest

The authors declare no conflicts of interest.

## Data Availability

The data that support the findings of this study are available on request from the corresponding author. The data are not publicly available due to privacy or ethical restrictions.
